# Catalytic Methane Decomposition to Carbon Nanostructures and CO_x_-Free Hydrogen: A Mini-Review

**DOI:** 10.3390/nano11051226

**Published:** 2021-05-06

**Authors:** Ahmed Gamal, Kamel Eid, Muftah H. El-Naas, Dharmesh Kumar, Anand Kumar

**Affiliations:** 1 Gas Processing Center, College of Engineering, Qatar University, Doha 2713, Qatar; a.mohamad@qu.edu.qa (A.G.); muftah@qu.edu.qa (M.H.E.-N.); 2 Qatar Shell Research & Technology Center (QSTP), Doha 3747, Qatar; Dharmesh.kumar@shell.com; 3 Department of Chemical Engineering, College of Engineering, Qatar University, Doha 2713, Qatar; akumar@qu.edu.qa

**Keywords:** methane, metal catalysts, CO_x_-free hydrogen, carbon nanostructures, carbonaceous catalysts

## Abstract

Catalytic methane decomposition (CMD) is a highly promising approach for the rational production of relatively CO_x_-free hydrogen and carbon nanostructures, which are both important in multidisciplinary catalytic applications, electronics, fuel cells, etc. Research on CMD has been expanding in recent years with more than 2000 studies in the last five years alone. It is therefore a daunting task to provide a timely update on recent advances in the CMD process, related catalysis, kinetics, and reaction products. This mini-review emphasizes recent studies on the CMD process investigating self-standing/supported metal-based catalysts (e.g., Fe, Ni, Co, and Cu), metal oxide supports (e.g., SiO_2_, Al_2_O_3_, and TiO_2_), and carbon-based catalysts (e.g., carbon blacks, carbon nanotubes, and activated carbons) alongside their parameters supported with various examples, schematics, and comparison tables. In addition, the review examines the effect of a catalyst’s shape and composition on CMD activity, stability, and products. It also attempts to bridge the gap between research and practical utilization of the CMD process and its future prospects.

## 1. Introduction

**Figure 1:**
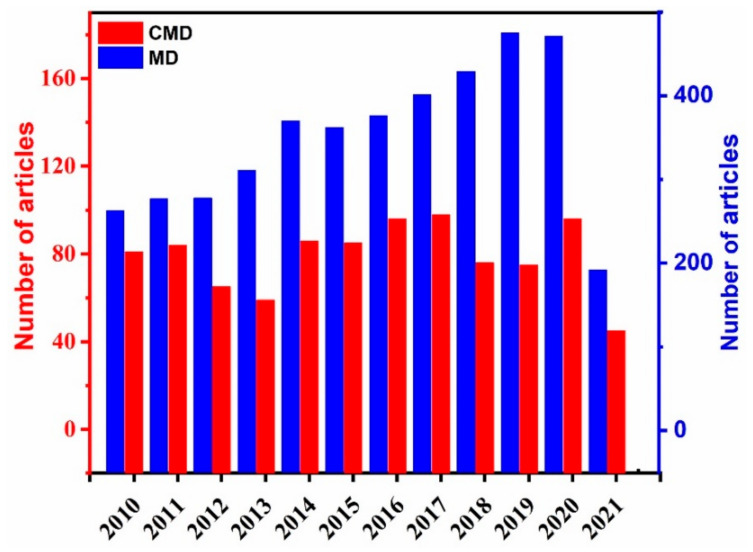
The chart displays the publications on catalytic methane decomposition (CMD) and methane decomposition (MD). The data were collected from Scopus using the keywords “catalytic methane decomposition” and “methane decomposition” between 2010 and 2021.

Methane is a powerful greenhouse gas and is of great importance in power generation, hydrogen production, and methanol production. Catalytic methane decomposition (CMD) is one of the key areas of investigation as it splits natural gas directly into hydrogen and solid carbon. Hydrogen is an environmentally benign fuel with high heating value and CO_x_-free emission, whereas carbon has many industrial applications such as metal extraction, water purification, and pharmaceuticals. In addition, reducing greenhouse gases (GHG) in the atmosphere is of great importance in various industrial and environmental remediation applications [1,2,3,4]. Conventionally, hydrogen is produced through steam reforming of methane, auto thermal reforming of methane, water splitting, biomass, and coal gasification with varying levels of efficiency and productivity [5,6,7]. Unlike these methods, CMD possesses certain advantages, such as the production of relatively COxfree hydrogen, lower CO_2_ emission, and feedstock [8]. Methane is also considered the best source for hydrogen production than other hydrocarbons due to its high hydrogen/carbon ratio of 4/1 and the fact it can be easily stored and transported [9]. Catalysts play a vital role in the CMD process. There are extensive reports on CMD using various catalysts such as transition metals (Fe, Ni, Cu, and Co), noble metals (Pd, Au, Pt, and Ir), metal oxides, and carbon (graphene and carbon nanotubes) and their composites [8,10,11,12,13,14,15,16,17]. Overall, the shape, composition, size, and surface features of the catalysts are the main factors determining catalytic performance towards CMD, which has attracted considerable attention in the last few years [18,19,20,21]. According to Scopus, more than 2000 articles have been devoted to CMD in the last five years (Figure 1). Some reviews were published recently focused on Ni-based catalysts as a function of support, loading amount, composition, and preparation method [22,23,24]. Likewise, another review focused on metal-based (e.g., Ni, Fe, noble metal) catalysts with a special focus on the type of reactors used for CO_x_-free hydrogen production [25]. Thereby, there is a critical need to highlight the recent advances in CMD, related catalysis, reaction conditions, and reaction products from a quick development view.

Unlike the previous reviews, this review highlights the CMD process focusing on the production of COx-free hydrogen and carbon nanostructures (nanotubes, nanosheets, and flakes) using transition metal-based catalysts (Fe, Ni, Co, and Cu) self-standing or supported on different oxides (SiO_2_, Al_2_O_3_, TiO_2_, and La_2_O_3_) along with their mechanisms. Carbon-based catalysts (carbon blacks, carbon nanotubes, coal char, and activated carbon) for CMD are also highlighted in addition to the effect of the catalyst morphology, composition, and reaction conditions on CMD activity, stability, and products. The whole review comprises various quantitative and qualitative analyses on the fabrication and characterization of catalysts alongside their parameters supported with various examples, schematics, and comparison tables for CMD. Overall, the review aims to serve as an important roadmap to facilitate future research and technology development in CMD.

### Catalytic Methane Decomposition (CMD)

Methane is a stable molecule that needs extremely high temperatures to decompose (Equation 1) without catalysts due to its high symmetric tetrahedral structure and sigma-bond. Meanwhile, in the presence of catalysts, the reaction can occur at temperatures as low as 500–750 °C [25,26,27,28,29,30,31,32,33,34].

CH_4_ → C + 2H_2_ (1)

One of the possible mechanisms of CMD includes the initial dissociation of methane on the catalyst surface followed by hydrogen release and diffusion of carbon atoms into catalyst particles and assembly of carbon atoms to form different nanostructures (e.g., fiber, rods, tubes) [35]. Mainly, the catalysts should initially be activated under high temperature to allow cleaves in the C-H bond of methane to release hydrogen and subsequent deposition of C atoms in the form of nanostructures. Notably, to avoid the formation of various hydrocarbons, the dehydrogenative coupling should be precluded during the CMD.

## 2. Metal-Based Catalysts

Metals used in the catalytic methane decomposition process are mainly transition metals, so the following commonly reported transition metals are investigated in more detail: cobalt (Co), iron (Fe), nickel (Ni), and copper (Cu).

### 2.1. Cobalt-Based Catalysts

Cobalt (Co) is a frequently studied catalyst for CMD, used in monometallic and bimetallic states, and prepared by various methods such as coprecipitation, wet impregnation, sol-gel, etc. Jana et al. [36] prepared cobalt-based catalysts by the precipitation method and reported the effects and impact of the precipitating agent on catalytic performance. Catalysts were obtained by reduction of cobalt oxide precursors in ethylene glycol and using three different precipitating agents: sodium carbonate, ammonium hydroxide, and urea. Catalysts obtained from precursors precipitated with Na_2_CO_3_ or CO(NH_2_)_2_ showed remarkable catalytic activity at lower temperatures, which in both cases was assigned to the smaller particle size and aggregation degree of the final metallic Co phase. Accordingly, using urea as a precipitating agent led to the catalyst with the highest hydrogen production at 600 °C after 12 h of time on stream. Likewise, it is worth mentioning that the catalyst prepared using Na_2_CO_3_ showed significant activity in this reaction even at temperatures as low as 400 °C.

**Figure 2:**
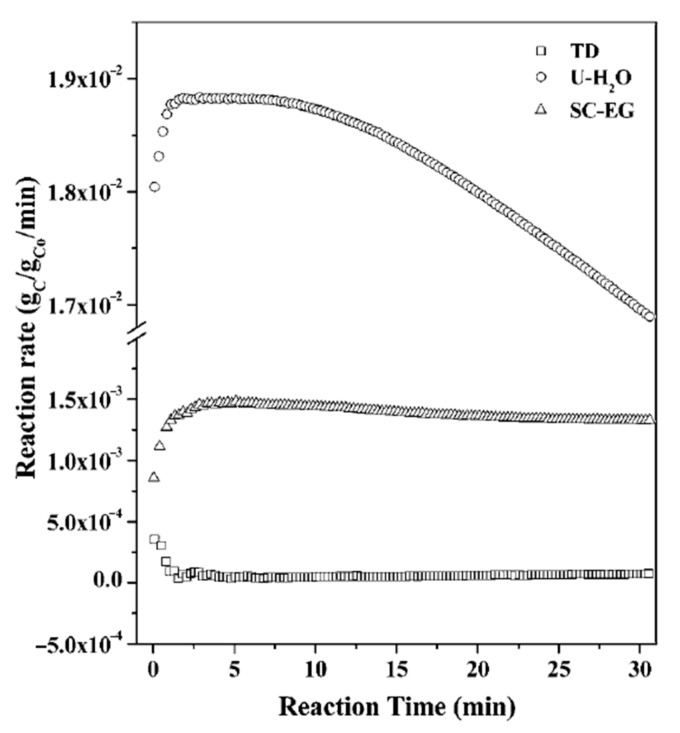
The catalytic activity of the three cobalt catalysts prepared by different methods: precipitation with urea in an aqueous medium (U-H_2_O), precipitation by using sodium carbonate in an ethylene glycol medium (SC-EG), and by thermal decomposition of cobalt nitrate (TD); reprinted with permission from ref. [37]. Copyright 2008 Royal Society of Chemistry.

In a further study, Jana et al. reported three cobalt-based catalysts prepared by precipitation with urea in an aqueous medium (U-H_2_O), precipitation using sodium carbonate in an ethylene glycol medium (SC-EG), and by thermal decomposition (TD) of cobalt nitrate [37]. Figure 2 presents the catalytic performance of these catalysts where the catalyst prepared by urea in an aqueous medium (U-H_2_O) showed the highest catalytic activity, followed by the catalyst prepared using sodium carbonate in ethylene glycol medium (SC-EG). In contrast, the catalyst prepared by thermal decomposition (TD) of cobalt nitrate showed the lowest reaction rate. Nonetheless, the U-H_2_O catalyst showed rapid deactivation over a 30 min time on stream (TOS) compared to the SC-EG and TD catalysts, which were stable during the same TOS. The authors concluded that the type of carbon formed not only depends on the method of preparation of the cobalt catalyst but also on the reducing agent used for pretreatment of the catalyst to obtain metallic cobalt. The authors reported the formation of graphene sheets only when the reduction was made in a methane environment followed by CMD on the catalyst. In contrast, the reduction in the hydrogen environment did not produce graphitic carbon.

Chai et al. [38] reported the effect of supports on the performance of cobalt-based catalysts by loading a cobalt catalyst on various supports such as alumina (Al_2_O_3_), silica (SiO_2_), zeolite (H-ZSM-5), ceria (CeO_2_), titania (TiO_2_), calcium oxide(CaO), and magnesium oxide (MgO). Reactions were carried out in a fixed-bed reactor and at two operating temperatures, 550 °C and 700 °C. Cobalt catalysts supported on alumina showed the highest catalytic activity compared to the other catalysts at 550 °C and 700 °C (Table 1 and Table 2). Al_2_O_3_ allowed the growth of smaller-sized graphitic carbon nanotubes on the CoO/Al_2_O_3_ catalyst at 700 °C compared to the catalysts that formed large-sized carbon nanotubes. Additionally, they studied the effects of promoters, such as nickel oxide (NiO), copper oxide (CuO), iron oxide (FeO), and molybdenum oxide (MoO), on the cobalt catalysts. Among the promoters, FeO and MoO promoted the CoO/Al_2_O_3_ catalyst to form high-quality thin-wall carbon nanotubes while none of the promoters enhanced catalytic performance (Table 3).

**Figure 3:**
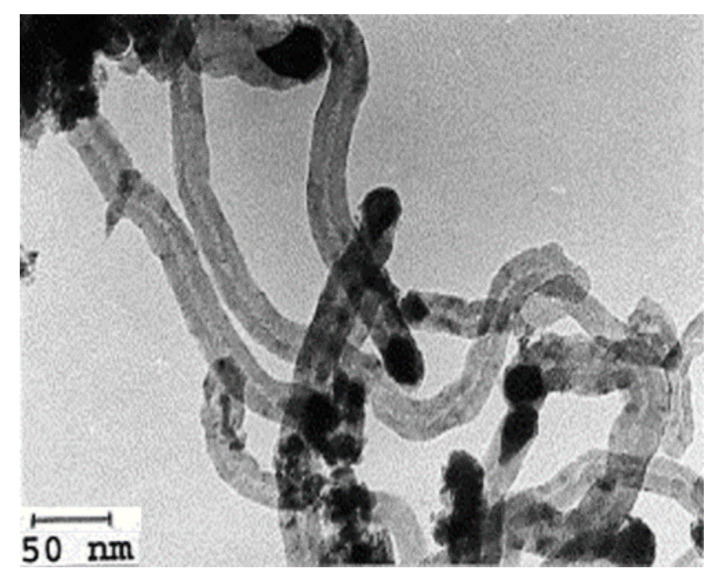
TEM image of spent Co/Al_2_O_3_ catalyst; reprinted with permission from ref. [39]. Copyright 1999 Elsevier.

Avdeeva et al. [39] reported a study of cobalt- and nickel-based catalysts supported on alumina. Catalysts were prepared using the coprecipitation method and reactions were performed in a vibrating flow reactor at 475–600 °C under the pressure of 1 bar. Both the catalysts showed nearly similar catalytic activity. However, on the type of carbon formed, they reported the formation of carbon filaments only from the cobalt alumina catalysts after 50 min reactions at 500 °C with a hollow-like core morphology as seen in the TEM image in Figure 3. The effects of support and metal loading amount on catalytic performance were also studied.

In many other reports, cobalt and nickel have been used as bimetallic catalysts in methane decomposition with different metals. Awadallah et al. [34] reported on the following bimetallic catalysts: 50%Ni/MgO, 25%Fe-25%Co/MgO, 25%Ni-25%Fe/MgO, and 25%Ni-25%Co/MgO. Among them, the 25%Fe-25%Co/MgO catalyst showed the highest catalytic performance with a more than 80% hydrogen yield over 550 min TOS [34]. Although many the researchers used cobalt supported on various materials, Prabhas et al. [40] reported a study of unsupported cobalt catalysts prepared using the Pechini method. The preparation method and activation process affected the morphology, redox properties, and catalytic activity. Co-based catalysts reduced by methane exhibited better catalytic activity and a higher carbon yield than those reduced by hydrogen.

### 2.2. Iron-Based Catalysts

Iron is one of the primary transition metals used in the catalytic methane decomposition process, where monometallic, bimetallic, and mixed iron-based catalysts were reported to be active in methane thermal activation.

#### 2.2.1. Monometallic Iron Catalysts

Zhou et al. [41] reported a study on the effect of iron loading in the preparation of catalysts by varying the amount of Fe starting from 0 wt.% Fe (only alumina support) to 100 wt.% Fe (unsupported). Catalysts were prepared using the fusion method and tested in a fixed-bed reactor at 750 °C under atmospheric pressure for methane decomposition. From Table 4, 41% Fe-Al_2_O_3_ displayed the highest methane conversion of 80% with a TOF of 113.5 s^−1^ at 750 °C for 10 h. Using characterization techniques, such as in situ XRD and H_2_-TPR, the authors found that 41 wt.% loading of iron led to the highest amount of FeAl_2_O_4_ phase.

**Figure 4:**
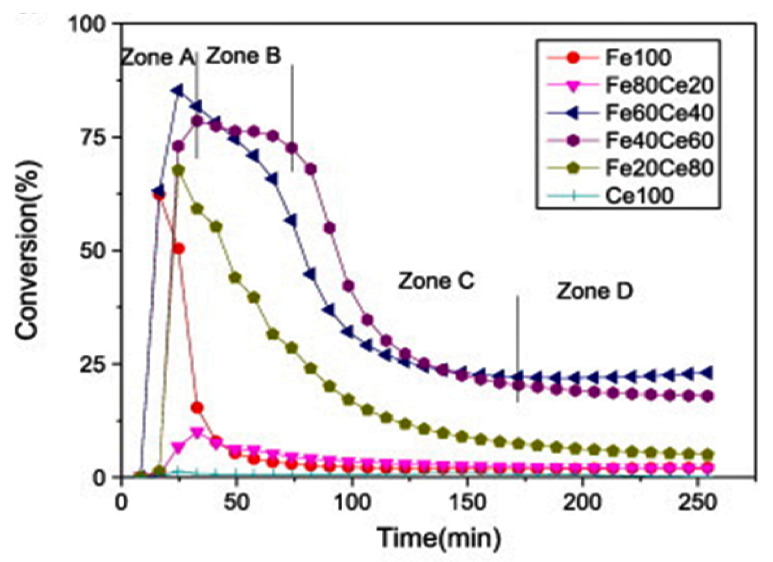
Effects of catalyst composition (different percentages of Fe and Ce) on methane conversion at 750 °C; reprinted with permission from ref. [42]. Copyright 2010 Elsevier.

Tang et al. [42] reported on a series of iron catalysts supported on ceria with various iron loadings (20 wt.% to 100 wt.% Fe), synthesized using the coprecipitation method, and tested in a conventional fixed-bed quartz reactor. None of the catalysts could sustain for a long time and suffered from severe deactivation. The 100%Fe, 100%Ce, and 80%Fe/CeO_2_ samples were the worst catalysts, deactivating within the first hour, while 20%Fe/CeO_2_ deactivated within 250 min. On the other hand, 40%Fe presented better activity and stability as the initial methane conversion was around 75% for the first 75 min then decreased to about 20% with slightly better stability after 120 min but with a slight deactivation behavior. The 60%Fe/CeO_2_ showed the highest catalytic performance as the catalyst displayed the highest iron dispersion and surface area. At 750 °C, it showed an initial methane conversion of about 85%, then dropped to 25% within the first 120 min and stayed stable until 250 min without deactivation. Figure 4 shows the catalytic performance at 750 °C for the various catalysts.

**Figure 5:**
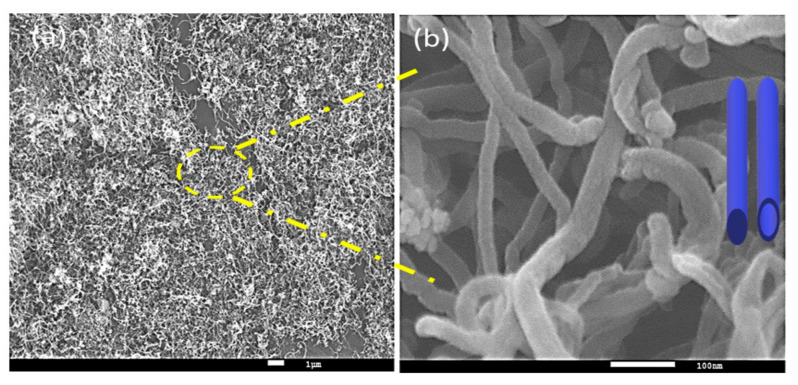
(**a**) SEM image of carbon nanotubes formed on 40%Fe/Al_2_O_3_ catalyst and (**b**) SEM image at a high magnification of the marked area in (**a**). The shape in (**b**) shows the proposed 3D model of carbon nanotubes. Reprinted with permission from ref. [28]. Copyright 2015 Elsevier.

Ibrahim et al. [28] reported a study of iron catalysts with different Fe loadings ranging from 15 wt.% Fe to 100 wt.% Fe supported on alumina (Al_2_O_3_) and prepared using the coprecipitation method. The hydrogen production increased with increasing iron loading, reaching 77.2% hydrogen yield using 60% Fe/Al_2_O_3_ at 700 °C for 4 h. On the other hand, catalysts with 15%, 25%, and 100% Fe loading showed poor catalytic activity and could not reach 20% hydrogen yield. The authors attribute the high catalytic activity to the right interaction between the metal and support that strongly affected the catalytic activity and carbon formation during the reaction. The carbon produced was characterized by using the SEM, which displayed the formation of filaments carbon nanotubes with different diameters over the spent 40%Fe/Al_2_O_3_ catalyst as shown in Figure 5.

Pudukudy et al. [43] reported on three monometallic catalysts prepared using the facile wet impregnation method: nickel(Ni), cobalt(Co), and iron(Fe) supported on sol-gel derived silica (SiO_2_). At 800 °C, the Ni-based catalyst showed 74% hydrogen yield, much higher than the Co- and Fe-based catalysts, which showed 43% and 46%, respectively, after 5 h TOS. SEM images indicated the existence of highly dispersed nanostructures of the metal oxides on the surface of the microsilica flakes. It also showed interwoven uniform multiwalled carbon nanotubes, irregular carbon particles with fruit-like structures, and multilayer graphene sheets over the Ni, Co, and Fe catalysts, respectively. The high graphitization degree, which was analyzed via Raman analysis, was responsible for the high catalytic performance of the catalysts. Table 5 shows a comparison among some of the promising monometallic iron-based catalysts and their catalytic performance in different operating conditions.

Wang et al. prepared Fe catalysts supported on different supports, including Al_2_O_3_, SiO_2_, and H-ZSM-5, reporting that those catalysts promoted the “base growth” carbon nanotube formation rather than the traditional “tip growth,” which enhanced the catalysts‘ regenerability [44]. Additionally, the interaction between the Fe and the support had an important role in the base growth mechanism. Fe/Al_2_O_3_ had a higher CMD activity than Fe/SiO_2_ and Fe/ZSM-5 due to the stronger interaction between Fe and Al_2_O_3_ than other supports. The quality of produced CNTs from the Fe/Al_2_O_3_ catalyst was about 96%. Qian et al. used 40 wt.% Fe on Al_2_O_3_ as an efficient catalyst for CMD in a fluidized bed reactor to investigate the effect of the reaction conditions [45]. The factors controlling the catalytic activity of the catalysts were the catalyst bulk density, particle size, minimum fluidization velocity, and the catalyst bed height. Using 20% H_2_-CH_4_ feed dilution was the best condition for CMD with a methane conversion of (70%) and quick activation time (5 min). The used Fe/Al_2_O_3_ catalyst was regenerated five times via carbon dioxide oxidation. It showed higher catalytic activity than the fresh ones as they contributed 75% methane conversion while the fresh ones showed only 70% [45]. That might be attributed to the formed catalytic bamboo-shaped carbon nanotubes, which can enhance the catalytic performance.

In another trial for optimizing the reaction conditions, Inaba et al. reported on Fe/Al_2_O_3_ catalysts provided by Süd-Chemie Catalysts Japan tested in a quartz reactor at 670–780 °C [10]. Increasing the space velocity led to decreasing the catalytic stability of Fe/Al_2_O_3_. The SEM and TEM images displayed the formation of highly crystalline and graphitic carbon nanofibers. Al-Fateh et al. investigated the effect of the preparation methods (i.e., impregnation, sol-gel, and coprecipitation) of Fe/Al_2_O_3_ on CMD performance [46]. The CMD activity was carried out at the same Fe loading amount (20 wt. %) on Al_2_O_3_ under the same conditions. The Fe/Al_2_O_3_ catalyst prepared using the impregnation method showed the highest catalytic performance compared to the other catalysts, which is plausibly attributed to the ability of the impregnation method to create adequate active sites on the surface of the catalyst.

Keller et al. reported on the effects of a spray-dried 10 wt.% Fe_2_O_3_/Al_2_O_3_ catalyst provided by Ohtsuka Ceramics Inc., Japan in a fluidized bed reactor [47]. The authors claimed that this catalyst could accommodate the formed carbon in its pores leading to less deactivation by the poisoning carbon. The activity of this catalyst regularly decreased with increased carbon formation on its surface. However, when the carbon produced was limited to below 10 wt.%, the stability of the catalyst was maintained. Hence, this developed catalyst can be helpful in more scale-up studies [47]. Geng et al. investigated Fe_2_O_3_ catalysts in a micro fluidized-bed reactor at reaction temperatures of 750–900 °C in addition to testing the same catalysts in a fixed-bed reactor [48]. The micro fluidized-bed reactor contributed a better CMD as the produced carbon nanotubes (CNTs) in the micro fluidized-bed reactor were more dispersed than that in the fixed-bed reactor and could not block the reactor. Konieczny et al. synthesized Fe catalysts from magnetite (Fe_3_O_4_) using methane or hydrogen as a reducing gas in a fixed-bed flow reactor at atmospheric pressures while heating from 800 to 900 °C [49]. Using methane induces the quick formation of Fe catalyst within 2 h at 900 °C without the need for a separate source of hydrogen at the plant site. Fe catalysts produced by methane allowed the complete CMD to form carbon nanofibers and hydrogen while Fe formed by hydrogen could not form carbon nanofibers [49]. Meanwhile, catalysts reported elsewhere only allowed for CMD (81%) while Fe produced by methane promoted complete CMD (100%) alongside maintaining the activity for 75 h. Al-Fateh et al. investigated the effects of WO_3_ and La_2_O_3_ on the catalytic performance of the Fe catalysts 20 wt.% Fe/ZrO_2_, 20 wt.% Fe/WO_3_-ZrO_2_, and 20 wt.% Fe/La_2_O_3_-ZrO_2_ prepared using the impregnation method and tested at a temperature of 800 °C [50]. The addition of WO_3_ to the support enhanced the catalytic performance in terms of CH_4_ conversion, H_2_ yield, and stability.

#### 2.2.2. Bimetallic Iron Catalysts

As iron is a promising metal in the catalytic decomposition of methane, many researchers have investigated the catalytic performance of iron in the presence of other elements by synthesizing bimetallic iron catalysts. Raney-type Fe-Cu catalysts, prepared from Me-Al alloys (Me = Fe or Cu), presented better stability than the monometallic Raney Fe catalysts [51]. This was due to the formation of incipiently alloyed Fe-Cu that helped in decreasing deactivation by encapsulating the carbon. Avdeeva et al. [52] reported on bimetallic iron–cobalt catalysts supported on alumina (Fe-Co/Al_2_O_3_) prepared using the coprecipitation method with different Fe-to-Co ratios and tested in a vibrating flow quartz reactor. They compared them to monometallic iron catalysts; as seen in Table 6, the bimetallic iron catalysts showed a higher methane conversion and stability at 625 °C. The catalysts prepared using the coprecipitation method showed better catalytic performance than catalysts prepared using the impregnation or precipitation method. The catalyst prepared using the coprecipitation of an aqueous solution of salts with NH_4_OH as a precipitant, calcined at 450 °C for three hours, and reduced at 580 °C for five hours showed the highest catalytic conversion of methane among the other coprecipitated catalysts.

Ayillath et al. [53] also reported on a comparative study about the catalytic activity and stability of monometallic and bimetallic iron catalysts (Fe, Ni, and Co) prepared using the dry impregnation method and supported on silica (SiO_2_). The authors supported the previous conclusion [53] that bimetallic catalysts showed better activity and stability than monometallic catalysts. Authors attributed the higher catalytic activity to the crystallite size of the bimetallic catalysts, which were found to be smaller than the monometallic ones, thus increasing the number of active sites and causing higher catalytic activity. The improved catalytic stability was attributed to the formation of an alloy in the bimetallic catalysts that prevented agglomeration and retained the catalyst stability. Additionally, Pudukudy et al. [54] reported a similar conclusion using Fe, Ni, and Co on a mesoporous silica support, attributing the high activity and stability to the alloy formation in the case of the bimetallic catalysts. Co-Fe alloy particles actively participated in the reaction more than the Nickel-based alloys.

**Figure 6:**
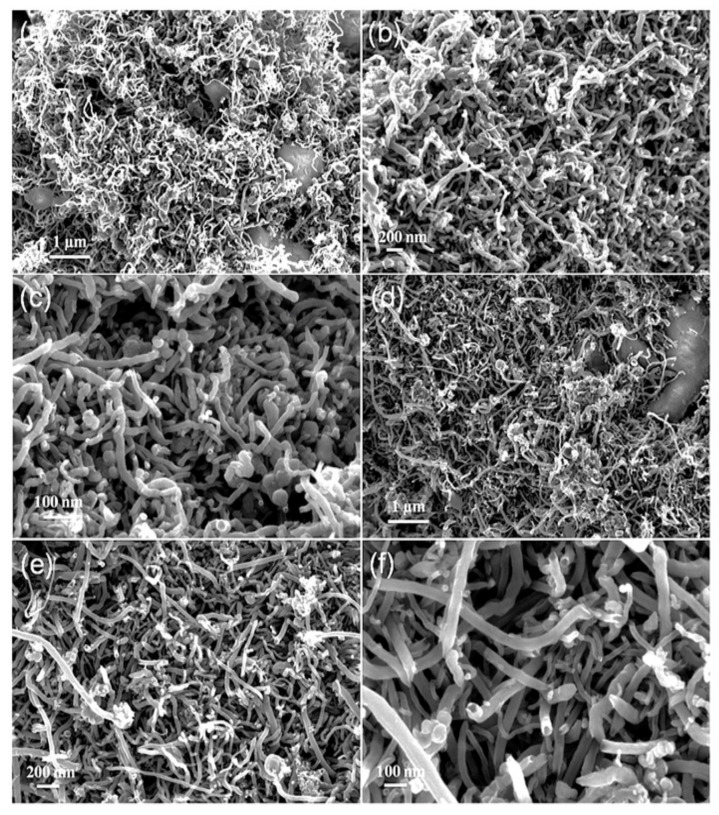
FE-SEM images of nanocarbon deposited over (**a**–**c**) Ni-Co/SBA-15 and (**d**–**f**) Ni-Fe/SBA-15; reprinted with permission from ref. [54]. Copyright 2015 Elsevier.

Regarding the carbon formation over the bimetallic catalysts, they studied it by using the FE-SEM technique and the images at different magnifications are presented in Figure 6 showing the carbon deposited on Ni-Co/SBA-15 (Figure 6a–c) and Ni-Fe/SBA-15 (Figure 6d–f). It was found that the catalyst surface was entirely covered with worm-like carbon nanotubes. The carbon nanotubes were thick, hollow, and contained opened tips.

Using promoters can enhance CMD significantly. The bimetallic Fe–Ni/Al_2_O_3_ catalyst was promoted with KCl-NiCl_2_ prepared using the molten salt approach, which resulted in high catalytic activity and stability for 1000 min TOS without showing any deactivation behavior [55]. The effect of the molten salt was attributed to the high wettability of the Fe-Ni alloy, which may have helped with the encapsulating of the carbon and precluded the deactivation [55]. Bimetallic Fe-Ni catalysts on a calcium silicate-Al_2_O_3_ support synthesized using the coimpregnation method for CMD at different temperatures from 600 °C to 800 °C showed that there was no direct relation between the surface area of the catalyst and the catalytic activity [55]. By contrast, the crystal structure of Fe and its loading amount had an obvious effect on CMD [56]. Shah et al. investigated bimetallic Fe-M (M = Pd, Mo, or Ni) catalysts supported on Al_2_O_3_ for CMD, which all showed higher activity than the monometallic Fe alone at 400–500 °C [57]. The catalysts were also tested above 900 °C where the produced carbon was graphitic films deposited everywhere in the reactor. All results indicated that integrating one or two metals with Fe was the key to enhancing CMD activity and the quality of the produced carbon due to the bimetallic effect along with the unique electronic effect and a high tolerance for the poisoning species or intermediates. Likewise, using multiple metal supports with Fe could substantially enhance the CMD as derived from the interaction of Fe with supports that tailor the activation of methane and avoid the oxidation process along with precluding adsorption of reaction intermediates or products.

### 2.3. Nickel-Based Catalysts

Nickel is a well-known catalyst in catalytic processes involving methane activation such as methane steam reforming and dry methane reforming. It is also reported to be active as a monometallic catalyst, bimetallic catalyst, and part of mixed metallic catalysts in CMD. The performance of nickel-based catalysts depends on loading percentages, alloyed metal, composition, preparation, support type, and activation method [23,58,59,60,61,62,63].

#### 2.3.1. Monometallic Ni Catalysts

Ermakova et al. [64] reported that they synthesized nickel catalysts on different supports, including impregnation of Ni oxide with SiO_2_, Al_2_O_3_, MgO, TiO_2_, and ZrO_2_, that worked as textural promoters and protected the metal particles against sintering. Catalysts were tested in a vibro-fluidized bed at 550 °C using a laboratory installation with a quartz flow reactor. Hydrogen and filamentous carbon were produced in different amounts depending on the nickel amount loaded in the catalysts. Moreover, regarding the effect of supports on the catalytic performance of nickel catalysts, the highest yield of carbon (375–384 g carbon per g nickel) was observed from the catalyst 96 Wt. % Ni/SiO_2_, which had particles with 10–40 nm average diameters. The effect of textural promoters (SiO_2_, Al_2_O_3_, MgO, TiO_2_, and ZrO_2_) on the catalyst performance was studied; the highest carbon yield was obtained with the silica.

Piao et al. [65] reported nickel supported on alumina catalysts prepared by the sol-gel method producing different amounts of hydrogen and carbon nanotubes, concluding that the catalytic activity depended on the nickel loading in the catalyst. The reduction and reaction conditions affected the morphology of the carbon formed. Kang et al. [59] reported on Ni catalysts prepared using the core-shell method from a single-step reaction of CO_2_ with NaBH_4_ at 1 bar with different loading amounts of nickel. Authors reported that the lowest loading of nickel (11%) in the catalysts showed better catalytic performance at 750 °C and 850 °C, as shown in Table 7. Catalysts with an 11%Ni loading had a higher hydrogen production rate than Ni catalysts with a loading of 13% or 19%.

Ziebro et al. [66] reported the effect of supports on Ni catalyst types formed on a Ni catalyst in CMD reaction. The authors used zeolite and silica as supports. They reported Ni/ZSM-5 catalysts, with a high silica ZSM-5 support, provided multiwalled carbon nanotubes, especially in low-temperature reactions (400–550 °C) with diameters from 8 to 63 nm and lengths from 60 to 413 nm. The growth of CNTs increased with increasing operating temperatures.

Using various supports, such as carbon [67], silica [68], and mixed La_2_O_3_/Al_2_O_3_ [69], with Ni-based catalysts enhanced CMD significantly. The CMD activity of Ni was augmented by doping with Ce, Mg, and Cu in the presence of sucrose as an addition agent [70,71]. Ashik et al. obtained nickel supported on silica (Ni/SiO_2_) using the coprecipitation cum modified Stöber method for CMD in a pilot plant [72]. The weight of the catalyst was the most effective factor among the other reaction conditions, such as the reaction temperature, which was classified as the second most effective factor controlling the reaction. The CMD performance depended on the amount of produced carbon formed in fishbone-like carbon nanotubes (CNT). Ni with different loadings (25, 40, 55, and 70 wt.%) supported on mesoporous spherical silica (Ni/SiO_2_) was prepared using the Stöber method for CMD [73]. The BET surface area of the Ni/SiO_2_ catalysts decreased with increasing Ni loading amounts due to the larger particle size and subsequent agglomeration at higher Ni loading amounts. Meanwhile, Ni/SiO_2_ (55 wt.%) showed the highest methane conversion of 54% at 575 °C among the other catalysts. However, a deactivation behavior was noticed during 300 min TOS.

Kuvshinov prepared NiO/Al_2_O_3_ catalysts in one step using solution combustion synthesis (SCS) with the assistance of hexamethylenetetramine (HMT) as a new active fuel with a specific fuel percentage (fuel coefficient of φ = 0.7) [74]. The as-synthesized NiO/Al_2_O_3_ catalysts were active in the CMD reactions and outperformed other catalysts published elsewhere. The results warranted using HMT as a fuel in the SCS method and could be extended to prepare other metal-based catalysts for CMD. Gubanov et al. [75] prepared three different catalysts to study the effects of the support structures on the catalytic behavior. The first was a Ni hydrotalcite catalyst prepared using the pH-controlled coprecipitation method. The second was a Ni ethylenediaminetetraacetate (EDTA) catalyst that was prepared in two steps: coprecipitation then thermolysis products were placed into a Na_2_[Ni–EDTA] solution and held there for 10 h, after which the the Ni–EDTA precipitate was washed, filtered, and dried at 120 °C. The third catalyst was a nickel catalyst supported on carbon nanotubes (Ni/CNT) prepared using the impregnation method. The addition of EDTA enhanced the catalytic performance of the Ni–EDTA catalyst as it sowed two temperature ranges of catalytic activity (550–650 °C and 700–850 °C) while the Ni/CNT catalyst did not show activity in the low temperature region (550–650 °C), which might be corresponded to the weak interaction with the carbon support. Xu et al. reported that the different locations of Ni species in HZSM-5 lead to different directions of methane reaction [76]. The supported Ni clusters could provide complete methane decomposition while the Ni-exchanged sites anchored at Brønsted acid sites may activate CH_4_ to CH_x_ species, which are required precursors to form aromatics. Shi et al. reported Ni–Al hydrotalcite catalysts were pre-reduced by H_2_ at 800 °C and tested at 500–700 °C. XRD, H_2_-TPR, and XPS showed that most Ni species are reduced to metallic Ni, which is the active phase leading to high catalytic activity [77]. Also, the carbon yield increased with increasing operating temperatures. Interestingly, the carbon formed at 500–550 °C were fishbone carbon nanofibers, while carbon formed at 600–650 °C were multiwalled carbon nanotubes.

#### 2.3.2. Bimetallic Ni Catalysts

**Figure 7:**
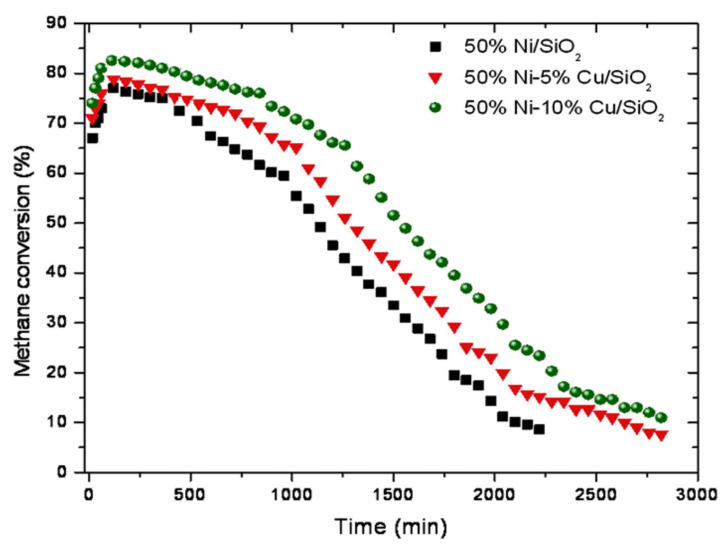
Catalytic performance of various Ni-Cu/SiO_2_ catalysts at 750 °C; reprinted with permission from ref. [79]. Copyright 2013 Elsevier.

Various bimetallic Ni-based catalysts, such as nickel with iron and nickel with copper, have been reported elsewhere [53,78,79,80]. Saraswat et al. [79] reported a comparative study explaining the difference between the catalytic performance of Ni-mono catalysts and Ni-bimetallic catalysts synthesized using the wet impregnation method and supported on silica. As reported, Ni loading and Cu promoter loading played a significant role in overall activity compared to monometallic Ni catalysts. As presented in Figure 7, higher loading of Cu (10%) resulted in the higher activity compared to Cu loading of 5% or no Cu loading. Incorporation of copper on nickel leads to an increase in the methane conversion/hydrogen yield. Authors interpreted that copper has a high affinity with carbon material, which inhibits carbon growth rate on the nickel catalyst and delays encapsulation of catalyst particles by carbon layers. Ni-Cu/Al_2_O_3_ bimetallic catalysts with different Cu/Ni ratios were prepared using the wet impregnation method for CMD [81]. The H_2_-TPR showed that adding 15 wt.% Cu to 50 wt.% Ni/Al_2_O_3_ caused a reduction shift toward lower temperatures and the XRD showed overlapping peaks of NiO and CuO indicating the formation of mixed oxides NixCu_(1−x)_O. The catalytic activity and stability of 15Cu-50Ni/Al_2_O_3_ were higher than monometallic Ni catalysts. NiMgAl mixed oxide catalysts were prepared using the precipitation method with various nickel nanoparticles ranging from 13.2 nm to 25.4 nm to determine the effect of nanoparticle size on the type of carbon product [82]. The carbon type depended on the Ni nanoparticle size significantly. Additionally, carbon nanotubes were prone to being deposited on NiMgAl with a larger Ni size while smaller Ni size allowed for carbon encapsulation. Torres et al. prepared bimetallic catalysts Ni-Cu/Al and Ni-Cu/Mg for CMD at 550, 600, 650, 700, and 750 °C [83]. Bimetallic Ni-Cu catalysts showed higher catalytic activity than the monometallic Ni in addition to the formation of fishbone carbon nanofibers. A hydrotalcite-based Ni–Mg–Al catalyst was prepared using the coprecipitation method with different nickel loadings (15, 40, and 65 wt.%) for CMD in a fixed-bed reactor [84]. Ni–Mg–Al containing 40 wt.% Ni showed the highest catalytic activity with about 80% methane conversion for 7 h.

The as-produced carbon nanofibers (CNFs) formed on the surface of Ni–Mg–Al generated active NiO species leading to more accessible active sites. Rastegarpanah et al. studied the effects of group VIB metals (Cr, Mo, and W) on a Ni catalyst (55 wt.% Ni/MgO) [85]. The subsequent catalysts were synthesized using the facile “one-pot” evaporation-induced self-assembly in ethanol and wetness impregnation method. The addition of the group VIB metals, particularly in the 5, 10, and 15 wt.% Cr to the Ni/MgO catalysts, enhanced CMD performance with methane conversions of 80, 87, and 75%, respectively, at 675 °C. The greater activity in the presence of Cr was attributed to the higher surface area and better reducibility. These results demonstrated the significant effect of combining one or two metals or support with Ni on the enhancement of CMD activity and the quality of produced carbon CO_x_-free hydrogen due to the multimetallic effect, electronic effect, promotion of the non-oxidative pathway, and high tolerance for the poisoning species or intermediates.

### 2.4. Copper-Based Catalysts

Copper is not a common monometallic catalyst for CMD; however, Ammendola et al. [86] investigated the effect of Cu on alumina using the wet impregnation method for CMD at 800 °C as a function of Cu loading. They reported that the low amounts of copper in the catalyst led to higher catalytic activity, as shown in Table 8, where the lowest copper loading catalyst (0.4Cu/Al_2_O_3_) contributed to the highest hydrogen production. In contrast, the catalyst with the most copper loading (8.4Cu/Al_2_O_3_) contributed the worst catalytic performance.

As mentioned earlier, copper has been used with iron and nickel as a bimetallic catalyst for CMD, affecting the catalytic activity and the textural properties of the catalyst and the kind of carbon produced [51,79]. Reshetenko et al. [87] reported on different Ni–Cu catalysts using copper as a promoter in the catalyst with various loading amounts (8%, 15%, 25, 35%, and 45%). The authors compared the results with Ni catalysts in the presence and absence of copper using a fluidized catalyst bed reactor to establish the effect of copper as a promoter in CMD. They interpreted that adding copper increases the yield of catalytic filamentous carbon (CFC) and controls both microstructural and textural properties, leading to an increase in the catalytic performance. As shown in Table 9, adding 8% of Cu to the Ni catalyst increased the methane conversion from 7% to 35% with an improved catalyst lifetime from 5 h to 9 h. The 15 wt.% of Cu was found to be ideal loading to obtain the highest conversion of methane and the catalyst’s stability.

Chen et al. [88,89] reported that adding copper to the nickel-based catalysts with specific amounts increased the catalyst’s activity and stability. Ni and Cu on Al_2_O_3_, with different compositions, were prepared using the coprecipitation method and tested in a fixed-bed reactor at 740 °C. Results of the initial catalytic activity are presented in Table 10. The 2Ni-1Cu-1Al_2_O_3_ catalyst stayed active for about 17 h with an initial methane conversion of about 55%. The catalyst composed of 15Ni-3Cu-2Al_2_O_3_ reached about 70% initial methane conversion but it was deactivated in about 4.5 h. Moreover, the authors suggested that the carbon growth mechanism is influenced by the reaction temperature.

**Figure 8:**
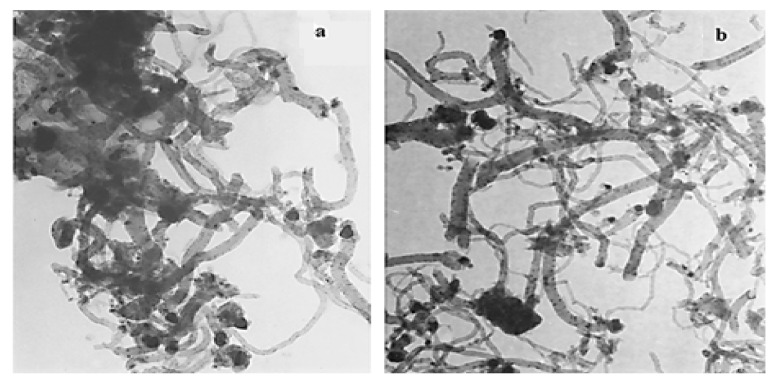
TEM images of carbon nanotubes obtained from methane cracking employing (**a**) pure Ni and (**b**) Ni–Cu; reprinted with permission from ref. [90]. Copyright 2010 Elsevier.

Regarding the effect of copper on the carbon formed during CMD, González et al. reported a study about the role of copper on unsupported nickel catalysts that were prepared directly by the physical mixing and thermal decomposition of the acetate parent salts and then used for generating carbon nanotubes through CMD [90]. The HR-TEM images showed the formation of carbon nanotubes with average diameter sizes between 50 and 60 nm on the pure Ni catalyst (Figure 8a). On Ni-Cu particles, CNTs with bimodal diameter distribution with values in the ranges of 20–30 nm and 60–70 nm were observed (Figure 8b). Accordingly, they suggested that copper induces the distribution of nickel nanoparticles without any aggregation after methane cracking; meanwhile, copper did not quantitatively improve the carbon formation.

## 3. Catalytic Supports

In general, studies have reported various supports play a significant role in improving catalytic performance in CMD reactions by influencing catalytic activity, lifetime, and carbon formation during the reaction [91,92,93].

### 3.1. Metal Oxide Supports

Takenaka et al. [91] reported a study on the effect of supports on Ni-based catalysts using eight different supports with the same nickel loading (25 wt.% of the catalyst). The authors concluded that Ni species were present as crystallized Ni metal particles in the active catalysts. By contrast, in the inactive catalysts, Ni species were present as nickel oxides, suggesting the formation of oxide between Ni and the support(s). The lifetime of the catalyst depended on the pore structure of the support. Silica without pore structure was the best support for the Ni catalysts, contributing the highest catalytic activity and the most extended lifetime among the other different tested supports, SiO_2_ (Cab-O-Sil), TiO_2_ (JRC-TIO-4), graphite, ZrO_2_ (JRC-ZRO-1), MgO·SiO_2_ (JRC-SM-1, MgO: 29.1 wt.%), MgO (JRC-MGO-1), SiO_2_·Al_2_O_3_ (JRC-SAH-1, Al_2_O_3_: 28.6 wt.%), and Al_2_O_3_ (JRC-ALO-4). All catalysts were prepared using the conventional impregnation method and tested under the same conditions (500 °C) and atmospheric pressure. The silica (SiO_2_) support showed the highest catalytic activity while the magnesia support (MgO) was the worst. Other supports were intermediate between these two, as shown in Table 11.

Takenaka et al. [92] reported that the support types influenced the catalytic activity and lifetime of cobalt-based catalysts. The study included four different supports: magnesia (MgO), alumina (Al_2_O_3_), silica (SiO_2_), and titania (TiO_2_) with 20% cobalt loading for each catalyst (20 wt.% Co) under the same reaction conditions at 500 °C. As shown in Table 12, the catalyst supported on alumina (20%Co/Al_2_O_3_) achieved the best catalytic activity and stability among the four catalysts with an initial methane conversion of 9% and the longest lifetime of 350 min of TOS. The magnesia-supported catalyst (20%Co/MgO) contributed an initial methane conversion of about 7% with a shorter lifetime of nearly 270 min of TOS. On the other hand, catalysts supported on silica and titania (20%Co/SiO_2_ and 20%Co/TiO_2_) contributed lower methane conversions and very low lifetimes. The catalytic activity and stability of the four catalysts followed the order of Co/Al_2_O_3_ > Co/MgO > Co/TiO_2_ > Co/SiO_2_. Co/Al_2_O_3_ had a smaller particle size than the other supports with an average size of 10–30 nm, leading to high conversion and longer TOS activity.

Chai et al. [38] reported the vital role of supports by studying cobalt-based catalysts with the same Co loading (10 wt. %) but supported on various types of materials (as presented in Table 13). These materials were tested under the same conditions at two different operating temperatures, 550 °C and 700 °C. The authors presented the effect of support on the catalytic activity in short-term reactions (0.5–2.0 h), concluding that Al_2_O_3_ support was more effective than other supports in enhancing cobalt catalytic activity at 700 °C. Simultaneously, silica performed better at the lower temperature (550 °C), as shown in Table 13.

**Figure 9:**
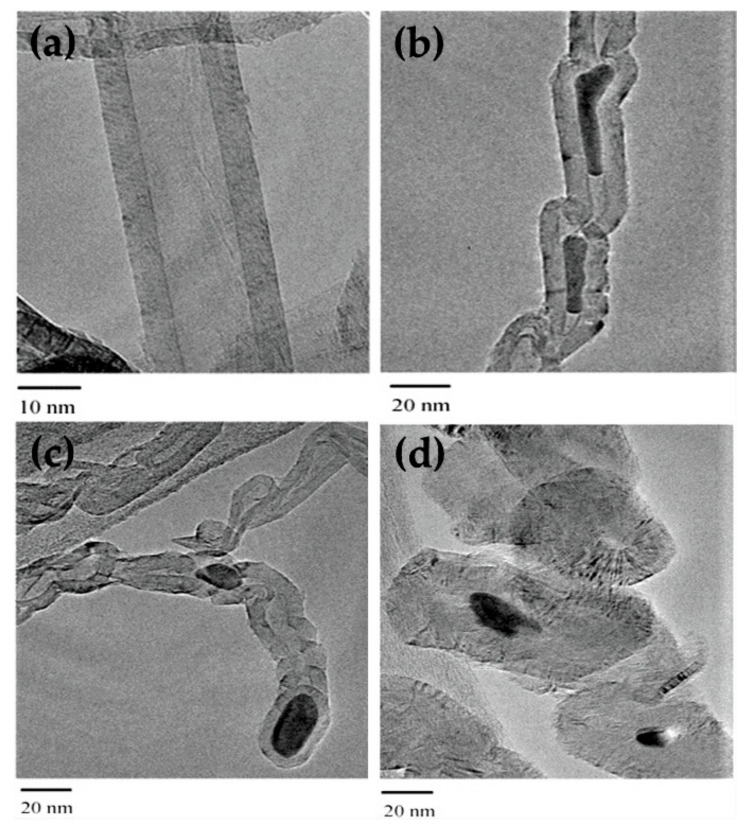
TEM images of different types of carbon formed on spent catalysts: multiwalled carbon nanotubes (**a**), chain-like carbon with cells filled by iron species (**b**), chain-like carbon similar to that of the Fe_2_O_3_/Al_2_O_3_ catalyst (**c**), and spherical carbon units without a hollow structure and iron species were sometimes found in the units (**d**); reprinted with permission from ref. [94]. Copyright 2004 Elsevier.

Silva et al. [93] reported a comparative study for cobalt catalysts on three different support materials: silica, alumina, and niobium oxide with the same Co loading (10 wt. %). Catalysts were prepared using the incipient wetness impregnation method with 10 wt. % of cobalt and were tested in a continuous quartz microreactor under the atmospheric pressure at 450 °C. Authors reported that Co/SiO_2_ showed the best catalytic activity with the highest methane conversion among the three catalysts, increasing activity with the reaction time. The enhancement in catalytic performance could be due to the reduction of oxide particles that were not completely reduced during activation/pretreatment that was carried out at low temperatures of 300 °C and 500 °C in hydrogen. Takenaka et al. [94] reported that the carbon structure formed during catalytic methane decomposition depends on the type of support used. The authors reported a comparison between two iron-based catalysts supported on silica and alumina prepared using the conventional impregnation method and tested in a fixed-bed reactor at 800 °C under the same operating conditions. According to the HR-TEM image in Figure 9, Fe_2_O_3_/Al_2_O_3_ catalysts produced two types of carbon: multiwalled carbon nanotubes, as seen in Figure 9a, and chain-like carbon with cells filled by iron species, as observed in Figure 9b.

On the other hand, the Fe_2_O_3_/SiO_2_ catalyst also formed chain-like carbon, similar to that of the Fe_2_O_3_/Al_2_O_3_ catalyst as shown in Figure 9c; additionally, many spherical carbon units without a hollow structure and with iron species were found, as seen in Figure 9d. Such carbon units without a hollow structure could not be observed in the TEM images of Fe_2_O_3_/Al_2_O_3_. The formation of porous carbon was attributed to the interaction of α-Fe with SiO_2_.

### 3.2. Activated Carbon Supports

**Figure 10:**
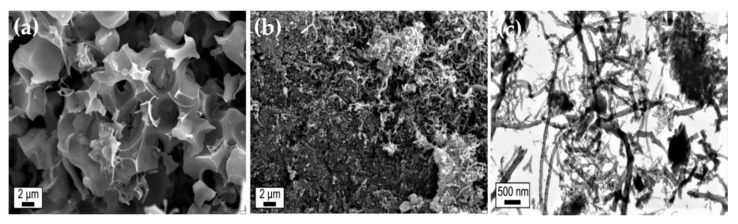
SEM micrograph of (**a**) a fresh 20% Pd/AC sample, (**b**) a 20%Pd/AC sample after the reaction at 850 °C, and (**c**) a TEM micrograph of a 20% Pd/AC sample after the reaction at 850 °C; reprinted with permission from ref. [95]. Copyright 2015 Elsevier.

**Figure 11:**
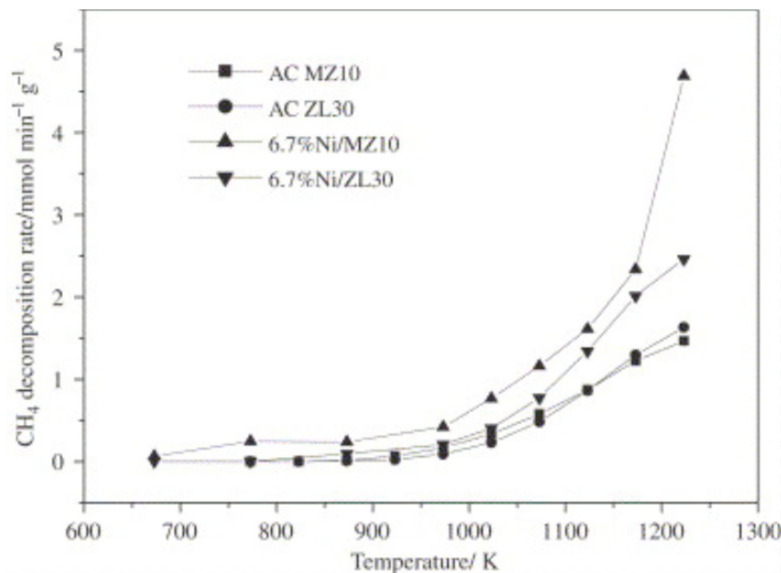
Catalytic activity tests at different operating temperatures of nickel catalysts supported on two commercial activated carbons, AC MZ10 and AC ZL30. reprinted with permission from ref. [96]. Copyright 2007 Elsevier.

Some studies have reported on carbon as a support, primarily activated carbon (AC), for CMD. Szymańska et al. [95] studied activated carbon as a support for different metals used in CMD. Activated carbon can work as a support beside working as a cocatalyst as well. In some cases, it may enhance the production of catalytic filamentous carbon, further increasing catalytic performance. The authors prepared three different catalysts of the metals platinum (Pt), palladium (Pd), and chromium (Cr) supported on activated carbon (AC) from ash wood biomass (Fraxinus excelsior L.). Catalysts were prepared using the incipient wetness impregnation method and tested at different temperatures (750 °C, 850 °C, and 950 °C) in a vertical fixed-bed quartz reactor. All catalysts worked in the methane decomposition reaction but only for a short time, suffering from a fast deactivation. In these cases, deactivation took place due to the formation of noncatalytic carbon, except in the Pd/AC catalyst, which showed high catalytic activity and stability due to the formation of catalytic filamentous carbon that further improved the activity without poisoning the catalyst. Their findings were confirmed by the SEM and TEM images taken of the fresh and spent catalysts tested at 850 °C, as shown in Figure 10. Bai et al. reported nickel catalysts supported on two commercial activated carbons (AC MZ10 and AC ZL30) prepared using the impregnation method and tested in a fixed-bed reactor [96]. The authors tested the original activated carbon as catalysts without metal (ACs only) for comparison. The 6.7%Ni/MZ10 catalyst was superior to the 6.7%Ni/ZL30, AC ZL30, and AC MZ10 catalysts at operation temperatures between 1000 and 1300 K, as shown in Figure 11. That higher catalytic activity was attributed to the formation of filamentous carbon with Ni metal on the tip, which increased the catalytic activity without poisoning the catalyst. However, new crystallite Ni_3_C in the spent catalysts was formed during the reaction that potentially may have caused deactivation of the catalyst.

## 4. Self-Standing Catalysts

Recently, several researchers have reported on the use of self-standing “unsupported” catalysts for methane decomposition reactions [97,98]. Pudukudy et al. reported porous NiO and Fe_2_O_3_ as catalysts for methane decomposition reactions without any support [97]. Catalysts were synthesized using the facile precipitation method and tested with pure methane in a tubular flow-cracking reactor made of stainless steel 2520 heated by an electric muffle furnace. The catalyst powder was packed in the middle of the reactor using thermal quartz wool. The two catalysts successfully showed high catalytic activity and even good stability at different operating temperatures (600 °C, 700 °C, and 800 °C). Additionally, NiO catalysts provided higher catalytic activity while Fe_2_O_3_ catalysts provided better stability due to their high carbon diffusion coefficient compared to the nickel catalyst.

**Figure 12:**
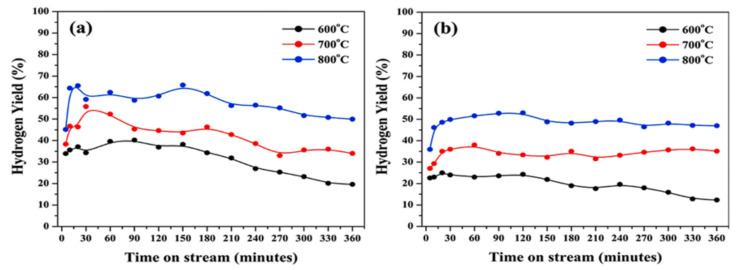
Catalytic activity tests carried out using (**a**) a NiO catalyst and (**b**) a Fe_2_O_3_ catalyst; reprinted with permission from ref. [97]. Copyright 2016 Elsevier.

Moreover, the NiO catalyst produced carbon nanochunks, and the Fe_2_O_3_ catalyst produced multilayer graphene sheets. At operating temperatures of 600 °C, 700 °C, and 800 °C, the two catalysts showed good stability for 360 min, as shown in Figure 12. Furthermore, Lua et al. reported on unsupported NiO and NiO-CuO catalysts for CMD reactions [98]. In the temperature range of 500 °C to 750 °C, the two catalysts showed high catalytic activity, particularly Ni–Cu catalysts, which reached about 80% methane conversion at 750 °C and good stability at the other operating temperatures. Authors attributed that high catalytic activity to the carbon nanofibers formed, which worked as support, taking away the catalyst particles and preventing them from sintering.

## 5. Carbon-Based Catalysts

**Figure 13:**
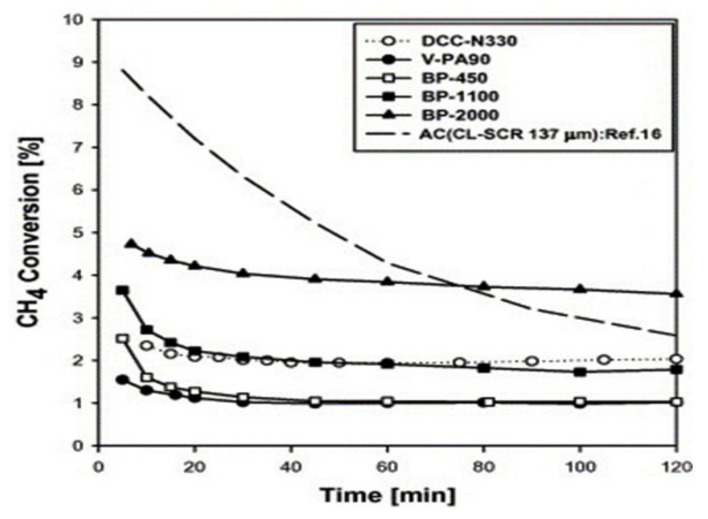
Catalytic performance of the carbonaceous catalysts at 850 °C with trade names CB-N330 (loose black), Vulcan PA90, Black Pearls 450, Black Pearls 1100, Black Pearls 2000, and another activated carbon catalyst, AC (CL-SCR 137); reprinted with permission from ref. [101]. Copyright 2004 Elsevier.

Many types of catalysts have been used in the catalytic decomposition of methane; however, coking remains the main challenge leading to catalyst deactivation. Therefore, researchers thought about using the carbon itself as a catalyst in the methane decomposition reaction to avoid carbon poisoning. Many carbon materials, such as mesoporous carbon, carbon blacks, carbon nanotubes, activated carbons, and coal char, were investigated as catalysts for methane decomposition reactions. Many studies on carbon-based catalysts have been reported; however, catalysts’ deactivation is still a grand challenge [99,100]. Lee et al. tested five commercial carbon blacks as catalysts for the methane decomposition reaction at different operating temperatures in a vertical fixed-bed reactor with the trade names CB-N330 (loose black), Vulcan PA90, Black Pearls 450, Black Pearls 1100, and Black Pearls 2000 [101]. The authors reported stable catalytic activity at all operating temperatures (850 °C to 1050 °C) despite carbon deposition. The carbon black catalysts (CBs) showed lower initial catalytic activity with high stability without deactivation as the carbon formed in the case of the CBs was catalytic carbon working as a catalyst and increasing the catalytic performance, as shown in Figure 13. In comparison, activated carbon catalyst AC (CL-SCR 137) showed higher initial catalytic activity but suffered severe deactivation during the two hours of the reaction carried out at 850 °C.

Serrano et al. investigated various types of carbonaceous materials as catalysts in the methane decomposition process: carbon blacks (CB), carbon nanotubes (MWNTs), mesoporous carbons (CMK), regular coke (Coke-1), re-carburizer coke (Coke-2), and graphite (GRAPH) [102]. Among these catalysts, the mesoporous carbons (CMKs) showed the highest catalytic activity in terms of the threshold temperature, defined as the initiation temperature of the CMD reaction, and detection of hydrogen. All the catalysts were tested at an operating temperature 1100 °C to ensure the highest catalytic activity and maximize hydrogen production. As presented in Table 14, the mesoporous carbon catalysts (CMK-3 and CMK-5) showed the highest catalytic activity with a maximum yield of H_2_ at the lowest threshold temperatures. On the other hand, catalysts made of coke (Coke-1) showed the minimum catalytic activity with the lowest yield of H_2_ at the highest operating temperature of 950 °C. The enhancement in the catalytic performance of the CMK catalysts was attributed to the abundant defects and mesoporous structure. The authors suggested that these defects were the main active sites for the decomposition of methane over carbon-based catalysts. The highest activity was exhibited by the carbonaceous catalysts with high defect concentrations present in the mesoporous carbon catalysts (CMKs).

As a trial for preparing a low-cost catalyst and understanding the methane decomposition reaction, Bai et al. tested coal char catalysts from the lignite [103]. Catalysts were tested in a fixed-bed reactor in a temperature range of 750 °C to 950 °C where acceptable but not high catalytic activity was reported without stating the stability of those catalysts. However, after characterizing the fresh and used catalysts, they noticed a decrease in surface area, pore volume, and micropore volume, along with an increase in the average pore diameter. Based on this, they proposed that the decomposition of methane occurred mainly in the micropores. A recent study reported on co-combined activated carbon (AC) with a carbon black (CB) used in the methane decomposition. Yang et al. [104] reported a different path of using hybrid AC-B with different compositions for CMD in a fixed-bed reactor at operating temperatures of 800 °C, 850 °C, and 900 °C compared to bare BC-B, AC, and CB. The AC was made from coconut shell and CB was acetylene black. Both were mixed in water via mechanical mixing with different ratios (denoted as AC0.25CB0.75, AC0.75CB0.25, and AC0.5CB0.5), then ultrasonically agitated for 30 min. Additionally, the researchers tested AC and CB alone without mixing and the result of the mixed catalysts were compared to them. The catalytic activity of the hybrid AC-CB was superior to its counterpart catalysts; however, AC-B was quickly deactivated.

**Figure 14:**
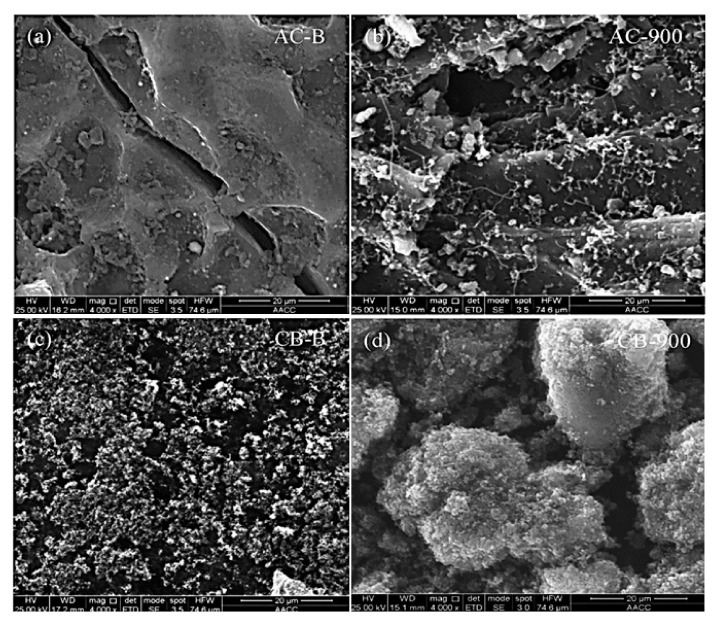
SEM image of (**a**) fresh activated carbon catalyst (AC), (**b**) spent AC catalyst after 500 min testing at 900 °C, (**c**) fresh carbon black catalyst (CB), and (**d**) spent CB after 500 min testing at 900 °C; reprinted with permission from ref. [104]. Copyright 2020 Elsevier.

Meanwhile, CB-B hybrid showed a stable catalytic performance that increased slowly over time. The SEM images of the catalysts before and after CMD displayed that, initially, AC-B was amorphous (Figure 14a), while CB–B was composed of small cluster-like particles (Figure 14c). After the CMD test, AC–B was covered in small-sized particles beside deposition of filamentous carbon in a nanofiber-like structure that plausibly originated from the metal impurities (Figure 14b). Although the deposited filamentous carbon is a highly active catalytic site, all catalysts were deactivated by the deposition of amorphous carbon, which blocked the micropores and encapsulated the metals. The carbon formed on CB-B after CMD were small-sized particles that agglomerated in the form of flake-like structures that were somehow active catalytic sites (Figure 14d). That was observed in the slow increase in the CMD by time over CB-B. Therefore, AC–B revealed the highest catalytic activity and durability relative to its counterpart catalysts due to the combination of the physicochemical and catalytic properties of AC and B beside their synergistic effect. This study may pave the way for the combination of various carbon-based catalysts for efficient CMD.

Kim et al. reported on a deactivation study for activated carbon catalysts made from coal for CMD in a fixed-bed reactor at an operating temperature of 850 °C [100]. The results showed a linear relationship between the amount of carbon formed and the deactivation of the catalyst. The uniform deposition of crystallites led to pore blocking and less accessibility to the active sites. Moreover, as an optimization study, they reported another relationship between space velocity and catalytic performance. Experiments were carried out using two different activated carbon catalysts (CCN-SCR and CL-SCR) at an 850 °C operating temperature with different space velocities. Based on the results, the authors concluded that lower space velocity resulted in higher methane conversion and vice-versa, attributing this to the residence time effect. Abbas et al. reported a kinetic and deactivation study for activated carbon materials manufactured from palm shells (ACPS) that was tested using a Mettler Toledo 850 Thermo-Gravimetric Analyzer and compared to commercial activated carbon [105]. Beside reporting that the ACPS contributed higher catalytic activity than the commercial AC, the catalytic activity of AC decreased linearly with increasing amounts of carbon formed on its surface. In a complementary pilot-scale unit study and by measurement of the surface properties of the fresh and spent activated carbon catalysts (ACPS), it was further validated that decomposition of methane occurs mainly in the micropores of the activated carbon [106].

Krzyzyński et al. reported on activated carbon samples prepared using Polish brown coal from “Konin” colliery as catalysts for the methane decomposition reaction at 750 °C, 850 °C, and 950 °C [107]. Samples were ground in a ball mill, sieved to the size of ≤0.2mm, and then subjected to acid demineralization. Most of the samples deactivated within 250 min, while a few showed better stability. Authors reported that the main challenge in large-scale use of the CMD was the catalysts’ gradual deactivation, which could be inhibited by employing a carbon-based catalyst with a large surface area with high pore volume. On the other hand, Kim et al. [108] reported no discernible relationship between the surface area and the activated carbon catalysts’ initial catalytic activity.

**Figure 15:**
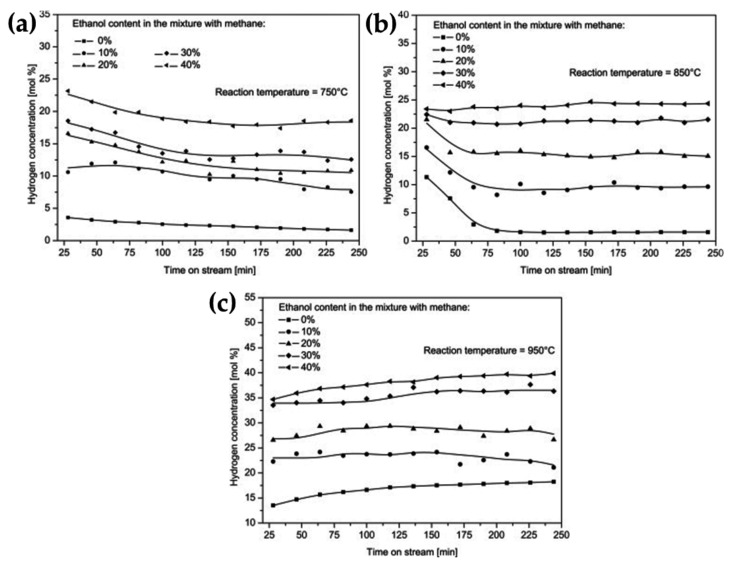
Effect of adding different amounts of ethanol to the CMD reaction at (**a**) 750 °C, (**b**) 850 °C, and (**c**) 950 °C; reprinted with permission from ref. [109]. Copyright 2012 Elsevier.

They tested commercial activated carbon from two sources, coconut shell and coal, at an operating temperature of 850 °C, compared the initial rate of methane decomposition against the surface area of the fresh AC catalysts, and concluded that there was no significant relationship between their CMD activity and the surface area of the catalyst. Rechnia et al. [109] conducted an optimization study to improve the stability and activity of AC catalysts. Authors reported cofeeding specific amounts of ethanol into the CMD reaction at 750 °C, 850 °C, and 950 °C to monitor the effect of ethanol on the reaction’s behavior, catalytic activity, and stability of the catalysts vs. a standard methane decomposition reaction. Best results were noticed with improved catalytic activity and stability with 40% ethanol in the feed at the three operating temperatures, as seen in Figure 15. It is worth noting there was CO_2_ formation, possibly due to the decomposition of ethanol in this study. Bai et al. reported on four different commercial activated carbons (DX40, CB10, MZ10, and ZL30) as catalysts for CMD in a fixed-bed quartz-tube reactor [110]. At 850 °C, all catalysts showed a deactivation behavior even though the initial catalytic activity was high for all catalysts. In other experiments, they tested only one catalyst (CB10) at different operating temperatures (750 °C, 800 °C, 850 °C, and 900°C) to conclude that deactivation was observed for all the temperatures. Additionally, the difference between the catalytic activity of AC catalysts was due to metal-contaminated ash resulting in increased catalytic activity and the formation of filamentous carbon. It is apparent in the SEM images of the used MZ10 catalyst (no ash contamination) had no filamentous carbon and used catalyst ZL30 (ash contamination) formed shiny filamentous carbon that contributed to the catalytic activity of the catalyst. Domínguez et al. studied the effect of the heating method on the performance of activated carbon catalysts during CMD and concluded that microwave heating (lower than or equal to 800 °C) resulted in higher methane conversion than electric heating [111]. The results showed the improved performance in microwave heating to the hot spots formation (microplasmas) inside the catalyst bed. As a summary of the factors controlling the catalytic methane decomposition process, Pinilla et al., in a kinetic study using carbonaceous catalysts, reported two competing points while using carbon-based catalysts for CMD studies [112]. A decrease in methane decomposition rate was observed due to the blocking of active sites by the deposited carbon, whereas an increase in methane decomposition rate could possibly be caused by catalytic carbon produced during the reaction.

The rapid deposition of coke over a catalyst’s surface blocked the active sites and poisoning of the catalysts reduced CMD activity. Thus, a suitable catalyst should counterbalance the CMD activity and stability. That can be achieved by the catalyst’s ability to initially activate the C–H bonds of methane, suppress the dehydrogenation and oxidation to avoid the generation of CH_3_·radicals, and subsequent formation of hydrocarbons [13,15,113]. To this end, a definitive study was conducted by Guo et al. to avoid the coking issue by converting methane into ethylene, aromatics, and hydrogen [13]. The authors reported an active catalyst composed of single iron sites embedded in a silica matrix that enabled methane conversion to ethylene, benzene, and naphthalene. They proposed that adjacent iron sites prevented the catalytic C–C coupling. Hence, coke deposition, resulting in methane conversion as high as 48.1% at 1090 °C with ethylene selectivity, peaked at 48.4%, and the total hydrocarbon selectivity exceeded 99% without producing any coke.

**Figure 16:**
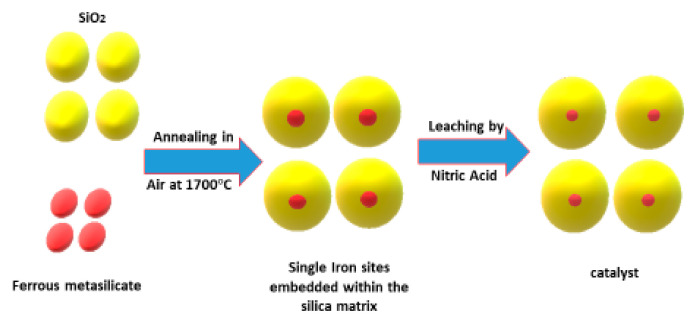
The preparation steps of the fused catalyst.

Moreover, a unique catalyst could be prepared using 0.5% loading of Fe on a silica support by fusing ferrous metasilicate with SiO_2_ at 1700 °C in air followed by leaching with nitric acid as illustrated in Figure 16. The single iron sites embedded in the silica matrix were observed using the HR-TEM technique. The authors reported a long-term stability test for the 0.5Fe@SiO_2_ catalyst at 1020 °C for 60 h. They achieved 32% methane conversion with around 55% selectivity for ethylene while producing benzene and naphthalene with 20% and 25% selectivity, respectively. They interpreted that the challenge laid in cleaving the first C–H bond while suppressing further catalytic dehydrogenation and avoiding CO_2_ generation or carbon formation. They reported that they could meet this condition by preparing the catalysts containing lattice-confined single iron sites in the silica matrix.

## 6. Conclusions and Future Prospects

### 6.1. Conclusions

In summary, this review assessed recent literature on the catalytic methane decomposition reaction for the production of relatively CO_x_-free hydrogen and carbon nanostructures, such as nanotubes, nanosheets, and flakes, using self-standing or supported metal-based catalysts, including Fe, Ni, Co, and Cu, on different supports. The effect of supports, including metal oxides (e.g., SiO_2_, Al_2_O_3_, and TiO_2_) and carbon-based supports (e.g., carbon blacks, carbon nanotubes, activated carbons), on CMD activity and stability was thoroughly reviewed and discussed. The review further elaborated on the effect of various parameters, such as temperature and catalyst composition, on the final products and yields.

The CMD activity of self-standing or supported Fe, Ni, Co, and Cu catalysts enhanced significantly using promoters or second metals in the form of alloys or core–shells. Using metal oxide supports also improved CMD performance as well as the COx-free hydrogen yield. Using multiple supports is preferred over one support due to the electronic effect and interaction with metal catalysts. Using elevated temperature (≥800 °C) is still preferred for CMD. Carbon-based catalysts (e.g., carbon blacks, carbon nanotubes, and activated carbons) with abundant defects or porosity enhanced the CMD and accelerated the reaction kinetics along with providing high stability.

### 6.2. Future Prospects and Research Trends

From the future perspective view, several challenges should be addressed in CMD to explore its potential for large-scale applications. Although great progress has been achieved in CMD, various challenges limit its practical economic application to produce CO_x_-free hydrogen and carbon materials. That includes the sluggish CMD reaction kinetics and the high operation cost and energy consumption (i.e., heating to elevated temperature) in addition to the low mass production of CO_x_-free hydrogen and carbon products. The isolation of carbon nanostructures from the catalyst surface is not only difficult but also poisoning to the catalyst and detrimental to CMD activity. Therefore, it is crucial to improve the CMD process at low operating temperatures, enhance the production yield of CO_x_-free hydrogen and carbon, and avoid catalyst poisoning in order for it to be feasible for industrial applications. The CMD process still needs significant development in terms of catalyst optimization, process design, and scale-up. To this end, although momentous achievements have been made in the fabrication of transition metal-based (e.g., Ni, Cu, CO, and Fe) catalysts, tailoring their morphology (e.g., porosity, dimension, accessible surface area, active sites, facets, and surface features) and composition (e.g., alloy, core-shell, and intermetallic) in one step at room temperature has not yet been reported.

Meanwhile, the preparation methods comprise multiple-step reactions and heating, which is a cumbersome process and allows segregation of metal precursors rather than mixing at the atomic level, devaluing the catalytic merits of the obtained catalysts. Thus, it is crucial to explore new green, simple, and one-step fabrication methods for controlling the size, shape, and composition of transition metal-based catalysts. Additionally, the catalysts should counterbalance the CMD activity and stability, which can be achieved by modulating the electronic effect of metal-based catalysts by alloying or forming intermetallic or core-shells with one or two more metals as well as using multiple transition metal oxides. The study of MXenes’ new classes of transition metal carbides, nitrides, or carbonitrides is among the hottest research trends nowadays; however, their gas conversion reactions are not highlighted enough relative to other energy and environmental applications [114,115]. Therefore, MXenes, with their unique physiochemical properties, multilayered, two-dimensional structure, and great electronic effects, could be promising catalysts for selective CMD under ambient conditions.

Noble metal-based catalysts are imminent with their outstanding catalytic performance for various catalytic reactions and CMD; therefore, porous multimetallic (e.g., bi-metallic and tri-metallic with or without support) noble metal-based catalysts, especially Pt-based [116,117,118,119,120], could enhance CMD activity, selectivity, and production quality or yield.

Carbon materials with specific morphological features (e.g., mono/multidimensional, porosities, multilayers, and branches) and compositional merits (e.g., doped atomically with metals/non-metals, single atoms, and functionalized with metal nanoparticles) [121,122,123,124,125,126,127] could enhance CMD significantly due to their low cost, outstanding physical–chemical–thermal stability, and massive active sites. 3D metal organic framework [128], carboxylated carbon [129], or carboxylated graphene [130] materials as well as biomass-derived porous nitrogenized carbon [131] and graphdiyne [132] can also be good candidates for CMD. Further theoretical modeling and artificial neural network modeling intelligence should be conducted to better understand the morphology/composition-related characteristics of the catalysts during CMD [133]. Theoretical studies are also needed to further develop novel catalysts for CMD.

## Figures and Tables

**Table 1:** The catalytic activity of cobalt catalysts on different supports at 550 °C and 1 atm [38].

Catalysts	CoO Loading (wt. %)	Methane Conversion * (%)	Duration (h)	Carbon Capacity (gc/gCoO) (%)
CoO/Al_2_O_3_	10	9.3	1.5	710
CoO/CaO	10	0.1	0.5	2.7
CoO/CeO_2_	10	3.0	0.5	70
CoO/H-ZSM-5	10	8.3	2	1245
CoO/MgO	10	0.2	0.5	4.1
CoO/SiO_2_	10	12.5	2	1337
CoO/TiO_2_	10	0.2	0.5	7.0

* Initial methane conversion.

**Table 2:** The catalytic activity of cobalt catalysts on different supports at 700 °C and 1 atm [38].

Catalysts	CoO Loading (wt. %)	Methane Conversion * (%)	Duration (h)	Carbon Capacity (gc/gCoO) (%)
CoO/Al_2_O_3_	10	6.5	1	223
CoO/CaO	10	0.7	0.5	14
CoO/CeO_2_	10	5.1	0.5	131
CoO/H-ZSM-5	10	4.7	0.5	122
CoO/MgO	10	0.4	0.5	13
CoO/SiO_2_	10	3.3	0.5	109
CoO/TiO_2_	10	1.4	0.5	45

* Initial methane conversion.

**Table 3:** The catalytic activity of cobalt catalysts including different promoters and tested at 700 °C and 1 atm [38].

Catalyst	CoO Loading (wt. %)	Promoter Loading (wt. %)	Time (h)	CH_4_ Conv. %
CoO/Al_2_O_3_	10	0	1	6.5
CoO–%CuO/Al_2_O_3_	8	2	0.5	2.9
CoO–FeO/Al_2_O_3_	8	2	0.5	5.2
CoO–MoO/Al_2_O_3_	8	2	0.5	6.3
CoO–NiO/Al_2_O_3_	8	2	0.5	5.3

**Table 4:** The catalytic activity of iron catalysts prepared via fusion method with different iron loadings and tested at 750 °C and 1 atm [41].

Catalyst	Time (h)	TOF of CH_4_ (S-1)
100%Al_2_O_3_	0.5	0.0
5% Fe-Al_2_O_3_	0.5	5.3
10% Fe-Al_2_O_3_	0.5	22.2
22% Fe-Al_2_O_3_	0.5	41.7
41% Fe-Al_2_O_3_	0.5	113.5
48% Fe-Al_2_O_3_	0.5	88.0
64% Fe-Al_2_O_3_	0.5	61.6
90% Fe-Al_2_O_3_	0.5	29.1
100% Fe	0.5	18.8

**Table 5:** The catalytic performance of promising iron monometallic catalysts that were tested in a fixed-bed reactor at different operating conditions collected from different papers [28,41,42,43].

Catalyst	Preparation Method	Temp. (°C)	Time (h)	Activity	Ref.
13.5%Fe/Al_2_O_3_	Fusion method	750	10	CH_4_ Conversion (80%)	[41]
41%Fe/Al_2_O_3_	Fusion Method	750	0.5	TOF of CH_4_(S-1) (113.5)	[41]
60%Fe/CeO_2_	Co-precipitation	750	1.5	CH_4_ Conversion (80–25%)	[42]
40%Fe/CeO_2_	Co-precipitation	750	1.5	CH_4_ Conversion (75–25%)	[42]
60%Fe/Al_2_O_3_	Co-precipitation	700	4	H_2_ Yield (77.2%)	[28]
80%Fe/Al_2_O_3_	Co-precipitation	700	4	H_2_ Yield (75%)	[28]
50%Fe/SiO_2_	facile wet impregnation	800	5	H_2_ Yield (46%)	[43]

**Table 6:** Comparison between the monometallic Fe and bimetallic Fe-Co catalysts prepared using the coprecipitation method and tested at 625 °C and 1 atm [52].

Catalyst	Time (h)	CH_4_ Conversion %
90Fe-Al_2_O_3_	7	5.2
85Fe-5Co-Al_2_O_3_	16.5	7.9
50Fe-Al_2_O_3_	23	4
50Fe-6Co-Al_2_O_3_	40	8

**Table 7:** The catalytic performance of Ni catalysts prepared using the core-shell method with different Ni loadings [59].

Catalyst	Temp. (°C)	H_2_ Production Rate (mmol/min g Ni)
19%Ni@C-B_2_O_3_	850	44
19%Ni@C-B_2_O_3_	750	41
13%Ni@C-B_2_O_3_	850	61
13%Ni@C-B_2_O_3_	750	47
11%Ni@C-B_2_O_3_	850	67
11%Ni@C-B_2_O_3_	750	47

**Table 8:** The catalytic activity of copper catalysts prepared using the wet impregnation method with different Cu loadings and tested at 800 °C and 1 atm [86].

Catalyst	H_2_/Cu (mol/mol)
8.4Cu/Al_2_O_3_	3.70
2.0Cu/Al_2_O_3_	6.16
0.4Cu/Al_2_O_3_	9.50

**Table 9:** The catalytic performance of Ni-Cu-Al_2_O_3_ catalysts prepared using the coprecipitation method and tested at 675 °C and 1 atm [87].

	Time (h)	CH_4_ Conversion (%)
90Ni-Al_2_O_3_	5	7
82Ni-8Cu-Al_2_O_3_	9	35
75Ni-15Cu-Al_2_O_3_	27.5	27
65Ni-25Cu-Al_2_O_3_	20	26
55Ni-35Cu-Al_2_O_3_	19.5	20
45Ni-45Cu-Al_2_O_3_	12	17

**Table 10:** The catalytic performance of Ni-Cu-Al_2_O_3_ catalysts prepared via coprecipitation method and tested at 740 °C and 1 atm [88,89].

Catalyst	Time (h)	Initial CH_4_ Conv. (%)
1Ni-1Cu-1 Al_2_O_3_	~5.5	~50
3Ni-3Cu-2 Al_2_O_3_	~6	~50
2Ni-1Cu-1 Al_2_O_3_	~17	~55
15Ni-3Cu-2 Al_2_O_3_	~4.5	~70

**Table 11:** Catalytic performance of Ni catalysts prepared via impregnation method and supported on various types of materials at 500 °C and 1 atm [91].

Catalyst	Lifetime (min)	H_2_ Production (mmol/g_cat_.)
Ni/SiO_2_	~380	1655
Ni/TiO_2_	~240	1153
Ni/graphite	~200	952
Ni/ZrO_2_	~150	195
Ni/SiO_2_·Al_2_O_3_	~120	64.2
Ni/Al_2_O_3_	Before 50 min	20.0
Ni/MgO·SiO_2_	Before 50 min	2.2
Ni/MgO	Before 50 min	0.75

**Table 12:** Catalytic performance of cobalt catalysts prepared via conventional impregnation method with different supports and tested at 500 °C and 1 atm [92].

Catalyst	Time (min.)	Initial CH_4_ Conv. (%)
20%Co/Al_2_O_3_	350	~8.5
20%Co/MgO	270	~7
20%Co/SiO_2_	110	~6
20%Co/TiO_2_	60	~3.5

**Table 13:** Catalytic performance of cobalt-based catalysts prepared via impregnation method with the same Co loading (10 wt.%) but supported on various types of materials [38].

Catalyst	Temp. (°C)	Time (h)	CH_4_ Conv. (%)
CoO/Al_2_O_3_	550	1.5	9.3
CoO/CaO	550	0.5	0.1
CoO/CeO_2_	550	0.5	3.0
CoO/H-ZSM-5	550	2.0	8.3
CoO/MgO	550	0.5	0.2
CoO/SiO_2_	550	2.0	12.5
CoO/TiO_2_	550	0.5	0.2
CoO/Al_2_O_3_	700	1.0	6.5
CoO/CaO	700	0.5	0.7
CoO/CeO_2_	700	0.5	5.1
CoO/H-ZSM-5	700	0.5	4.7
CoO/MgO	700	0.5	0.4
CoO/SiO_2_	700	0.5	3.3
CoO/TiO_2_	700	0.5	1.4

**Table 14:** A comparison to show the order of catalytic activity of tested carbonaceous catalysts from ref. [102].

Catalyst	Threshold Temperature (°C)	H_2_ Production (mmol/min. g_cat_)
AC	779	61
CB-bp	778	118
CB-v	795	93
MWNT-1	865	82
MWNT-2	870	80
GRAPH	910	23
Coke-1	950	3
CMK-3	744	219
CMK-5	753	428

## Data Availability

Data available on request from the corresponding author.
